# Identification of Hub Gene Characteristics and Immune Landscapes Across Aging Subtypes in Atherosclerosis: A Machine Learning‐Based Multi‐Omics Study With Experimental Verification

**DOI:** 10.1111/cbdd.70382

**Published:** 2026-08-27

**Authors:** Qiyu Fan, Kang Chen, Jibin Liu, Xun Diao, Zhuopeng Xia, Haizhong Yu, Haixia Zhu

**Affiliations:** ^1^ Department of Clinical Laboratory, Nantong Hospital of Traditional Chinese Medicine Nantong Hospital Affiliated to Nanjing University of Chinese Medicine Nantong Jiangsu China; ^2^ Department of Interventional Radiology The Affiliated Wuxi People's Hospital of Nanjing Medical University, Wuxi People's Hospital, Wuxi Medical Center Wuxi Jiangsu China; ^3^ Department of Cancer Research Center Tumor Hospital Affiliated to Nantong University, Medical School of Nantong University Nantong Jiangsu China; ^4^ Department of Clinical Laboratory West China Hospital Sichuan University Jintang Hospital, Jintang First People's Hospital Chengdu Sichuan China; ^5^ Department of General Surgery Nanjing Luhe People's Hospital Nanjing Jiangsu China

**Keywords:** aging, atherosclerosis, immune landscapes, machine learning, multi‐omics study

## Abstract

Aging is a major risk factor for atherosclerosis (AS), but the aging‐associated molecular characteristics and immune heterogeneity of AS remain incompletely understood. This study aimed to identify aging‐related hub genes and characterize immune landscapes across aging subtypes of AS using integrated bioinformatics and experimental validation. AS‐related candidate genes were found by overlapping DEGs identified by limma and key module genes identified by Weighted Correlation Network Analysis (WGCNA) based on the GSE100927 dataset. Consensus clustering based on aging‐related DEGs (differential expression analysis based on 125 aging‐related genes between AS and control) was performed to identify aging subtypes and characterize immune landscapes. Typical genes were picked out using four machine learning models: Support Vector Machine (SVM), Random Forest (RF), eXtreme Gradient Boosting (XGB), and Generalized Linear Model (GLM). The expression of central genes was verified through single—cell data analysis. Patients with AS were enrolled (*n* = 30), and atherosclerotic plaque and adjacent normal arterial tissues were collected. Quantitative Polymerase Chain Reaction (qPCR) validation was performed in atherosclerotic plaques and oxidized Low‐Density Lipoprotein (ox‐LDL)‐treated THP‐1 macrophage. The effect of crucial genes on ox‐LDL‐induced THP‐1 macrophage inflammatory response and foaming were validated by qPCR, ELISA and Oil Red O Staining. We identified 76 aging‐related DEGs and classified AS samples into two aging‐related subtypes (C1 and C2) with distinct immune infiltration characteristics. Machine learning analysis based on 43 candidate genes identified 5 hub genes: heat shock protein family B (small) member 7 (HSPB7), myelin expression factor 2 (MYEF2), dual specificity phosphatase 26 (DUSP26), tandem C2 domains, nuclear (TC2N), and phospholamban (PLN), whose expression patterns were further validated by single‐cell RNA sequencing. Moreover, TC2N and PLN were significantly downregulated in atherosclerotic plaque tissues. TC2N and PLN expressions in atherosclerotic plaque tissue of AS patient were significantly reduced. TC2N was significantly decreased in ox‐LDL‐treated THP‐1 macrophage. TC2N overexpression alleviated ox‐LDL‐induced THP‐1 macrophage inflammatory response but also alleviated cell foaming. This integrated bioinformatics and experimental study identified five hub genes (HSPB7, MYEF2, DUSP26, TC2N, and PLN) associated with AS. Experimental validation confirmed that TC2N is significantly downregulated in human atherosclerotic plaques and ox‐LDL‐treated THP‐1 macrophages, TC2N overexpression alleviates inflammatory responses and foam cell formation. These findings provide insights into the molecular mechanisms linking aging and immune dysregulation in AS and highlight TC2N as a potential regulator of AS progression.

## Graphical abstract

This integrated bioinformatics and experimental study identified five aging‐related hub genes (HSPB7, MYEF2, DUSP26, TC2N, and PLN) associated with atherosclerosis (AS). Experimental validation confirmed that TC2N is significantly downregulated in human atherosclerotic plaques and ox‐LDL‐treated THP‐1 macrophages, TC2N overexpression alleviates inflammatory responses and foam cell formation, which provide new perspectives for the diagnosis and treatment of AS.

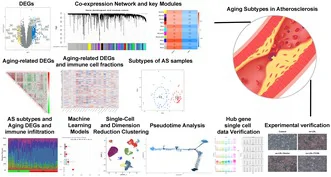

## 1 Introduction

Atherosclerosis, which is the main cause of most cardiovascular diseases, is a chronic inflammatory state and its incidence has risen significantly in recent years, posing a great danger to global public health. This pathological phenomenon mainly impacts medium—sized and large arteries and leads to acute cardiovascular incidents like unstable angina and acute myocardial infarction, making it the top cause of cardiovascular death across the world; well‐known risk factors are hypertension, diabetes, hypercholesterolemia (high LDL—cholesterol), smoking, and genetic tendencies. New research has shown that vascular senescent cells play an important part in the pathophysiology of atherosclerosis as these cells are involved in the development and instability of plaques (Ali et al. 2024) and thus cellular senescence is more and more regarded as a major risk factor for atherosclerotic cardiovascular disease.

Aging was defined as the gradual decrease in physiological functions within multiple tissues, signifying a basic biological process which ended in the emergence of chronic, age‐related illnesses and served as a main risk element for significant diseases such as cardiovascular problems, neurodegenerative conditions, metabolic disorders, and cancers (Abo Qoura et al. 2026). Recent investigations showed that aging was a chief factor in the rising rate of cardiovascular disease (Zhang, Yang, et al. 2025) and senescent cells were found to be crucial intermediaries and key points in the development of atherosclerosis (Tian et al. 2024). Key molecular mechanisms linking aging to atherosclerosis include p53/p21 and p16/Rb senescence pathways, the senescence‐associated secretory phenotype (SASP) characterized by pro‐inflammatory cytokines such as Interleukin‐6 and Interleukin‐1α, and specific aging‐related genes including GATA Binding Protein 4 (GATA4) and ABI3 (Xie et al. 2026; Kang et al. 2025; Fu et al. 2024). So, senolytic treatments which specifically got rid of senescent cells had come into being as a hopeful method to stop or reduce the progress of atherosclerosis by aiming at the harmful aging characteristics, and the use of gene expression analysis had become an essential means in modern biological research (Kalantari‐Dehaghi et al. 2025).

The swift progress of bioinformatics has greatly sped up biomedical research and the presence of vast amounts of high‐throughput data in public repositories makes it easier to figure out the underlying causes and pathological processes (Database Resources of the National Genomics Data Center 2026). While machine learning, because of its strong classification abilities, improves the dependability and diagnostic precision of disease evaluation systems and has been widely used to get feature representations from gene expression data (Maiti and Basak 2026; Jyothi et al. 2025). At present, combining bioinformatics analysis with machine learning and database verification is a crucial method for understanding molecular‐level pathology. Nevertheless, these methods are still not fully utilized in atherosclerosis research, leaving a large quantity of possibly valuable information untouched. Former studies showed that there was a tight connection between ferroptosis‐related genes, Glutathione Peroxidase 4 (GPX4) and transferrin receptor (TFRC), and the cell cycle regulators cyclin A2 (CCNA2) and cyclin dependent kinase 1 (CDK1), indicating that they might be potential therapeutic targets for atherosclerosis (Lu and Yu 2025).

Aging plays a critical role in the initiation and progression of atherosclerosis; however, the aging‐associated molecular mechanisms and immune heterogeneity underlying AS remain incompletely understood. Therefore, the present study aimed to systematically identify aging‐related key genes and characterize immune landscapes across distinct aging‐associated subtypes of AS through an integrated multi‐omics and machine‐learning framework. Furthermore, we sought to validate the expression patterns and biological functions of candidate hub genes using single‐cell transcriptomic data and experimental approaches. By elucidating the relationship between aging‐related molecular alterations and immune dysregulation in AS, this study provides new insights into the pathogenesis of AS and identifies potential molecular regulators associated with disease progression.

## 2 Materials and Methods

### 2.1 Raw Data Collection and Calibration

We downloaded a dataset called GSE100927 from a public database called Gene Expression Omnibus (GEO, https://www.ncbi.nlm.nih.gov/geo/) that can be used for this study. This set of data is based on the GPL17077 technology platform and includes 104 samples: 35 from healthy controls and 69 from AS patients. After obtaining the data, we first use the “Limma” toolkit in R statistical software (version 4.1.2) to uniformly process the data, with the aim of eliminating technical errors between different samples and making the data comparable. This processing is called ‘normalization’. The processed data is used for subsequent analysis. Meanwhile, according to the previous literature, we have successively obtained 125 genes related to aging (Saul et al. 2022).

### 2.2 Screening of Differential Genes Between AS and Control Group

The data after normalization were divided into case and control groups by means of the LIMMA R package, then DEGs were found and further filtered according to the standards of |log_2_ fold change| being no less than 1 and an adjusted *p*‐value being less than 0.05. Finally the significantly regulated DEGs were visualized by creating volcano plots and heat maps with the help of the ggplot_2_ and ComplexHeatmap R packages respectively.

### 2.3 Gene Set Enrichment Analysis (GSEA) of Differential Activation Pathways Between AS Group and Control Group

GSEA (https://www.gsea‐msigdb.org/gsea/) is a computational approach making use of carefully compiled molecular databases which include gene characteristics, genomic locations, and functional descriptions to spot statistically important enrichment trends. The ClusterProfiler R package was used to carry out Gene Ontology (GO, https://geneontology.org/) and Kyoto Encyclopedia of Genes and Genomes (KEGG, https://www.kegg.jp/) pathway enrichment analyses so as to clarify the biological processes being studied. In order to find out the differently activated pathways comparing experimental (AS) group with the control group, GSEA was performed; after that, we applied Gene Set Variation Analysis (GSVA) algorithm of GSVA R package to get a quantitative enrichment score for each biological process, which made it easier to evaluate the pathway activity among different sample groups.

### 2.4 Co‐Expression Network Construction

We built a WGCNA co‐expression network by means of the WGCNA R package, taking the GSE100927 dataset as the research focus and the Ankylosing Spondylitis (AS) group along with the normal control group as clinical features; the raw matrix data were standardized using the Fragments Per Kilobase Million (FPKM) method and genes having FPKM values less than 0.5 were removed to guarantee data quality; after eliminating outlier samples, the Pearson correlation coefficient was calculated to evaluate pairwise gene correlations; to maintain a scale‐free network structure, a soft threshold power (β) of 5 was chosen as it stresses strong correlations among genes and converts the similarity matrix into an adjacency matrix; then, we calculated the topological overlap matrix (TOM). At the same time, the dissimilarity matrix is obtained by 1‐TOM for subsequent hierarchical clustering; hierarchical clustering was carried out, based on the similarity of gene expression profiles, they are classified into corresponding modules, with a minimum module size (merge height) of 50; to further improve the module structure, the eigengene dissimilarity was assessed and a dynamic tree‐cutting method was used to combine closely‐related modules. Finally, 11 co‐expression clusters were recognized including the grey cluster, which represented genes that couldn't be allocated to any particular cluster.

### 2.5 Determine the Optimal Number of Subgroups for the Sample Through Consensus Clustering

Anosmia samples were divided into separate subgroups according to the expression patterns of age–related genes and the ideal quantity of clusters was decided through consensus clustering with up to nine clusters being assessed and the ultimate cluster solution was chosen when the consensus score surpassed 0.8 and all the analyses were carried out in the R programming environment.

### 2.6 Machine Learning Screening for AS Related Marker Genes

To identify robust hub genes associated with atherosclerosis, four machine learning algorithms, including Support Vector Machine (SVM), Random Forest (RF), eXtreme Gradient Boosting (XGB), and Generalized Linear Model (GLM), were applied to the candidate gene set. The predictive performance of each model was evaluated and compared, and genes consistently identified across multiple algorithms were considered potential hub genes. Subsequently, the selected hub genes were subjected to downstream validation and functional analyses.

### 2.7 Preprocessing and Dimensionality Reduction Clustering Analysis of Single Cell Data

The single‐cell RNA sequencing (scRNA‐seq) dataset GSE159677 (https://www.ncbi.nlm.nih.gov/geo/) (Alsaigh et al. 2022) comprises scRNA‐seq data from three human calcified atherosclerotic plaques and their matched proximal adjacent arterial tissues, generated on the Illumina Next‐Seq 500 platform. We analyzed this dataset with the Seurat v4 (Version 4.0.4) R package, and genes that were expressed in less than three cells as well as cells having the expression of fewer than 250 genes were excluded. Moreover, cells whose mitochondrial gene content exceeded 2% and ribosomal gene content surpassed 1% were also removed, after the quality control, principal component analysis (PCA) was utilized for dimensionality reduction which resulted in 50 principal components (PCs) each denoting a different cellular trait, then cells were grouped into separate clusters according to the similarity of these traits.

### 2.8 Pseudotime Reconstruction of Cell Differentiation Trajectories in AS Lesions

After annotating every single cell, we picked out the cell subtypes from the atherosclerotic (AC) group for the coming pseudo‐sequential inference and then utilized the “DDRTree” and “ReduceDimension” functions to sort out the differentiation states of all the cells and carry out dimensionality reduction on the AC group dataset. Later, the cells were projected onto a trajectory so that their differentiation trajectory could be visualized, which made it easier to simulate chondrocyte development and differentiation backed by a string of analytical processes and all the computational steps were carried out through the “plot_cell_trajectory” function in R software.

### 2.9 Patient Inclusion and Sample Collection

This retrospective study enrolled patients with AS from a center between January 2025 and June 2025, a total of 100 patients. According to the criteria of inclusion and exclusion, 50 patients with a history of other underlying diseases and 20 patients with incomplete clinical data were excluded. Finally, 30 patients were included in the final analysis. This research plan has been approved by Nantong Traditional Chinese Medicine Hospital (Approval No. 2025‐[004‐3]). Written informed consent was obtained from each participating patient. Atherosclerotic plaque and adjacent normal arterial tissues were collected from patients (Kanonykina et al. 2026).

The inclusion criteria were as follows: (1) All patients were confirmed by diagnostic biopsy or histopathology; (2) No prior history of chemotherapy, surgery, or other anticancer treatments; and (3) Availability of complete clinicopathological characteristics. Patients were excluded based on the following: (1) Diagnosis of a significant autoimmune disease; (2) Incomplete clinical data; and (3) Coexistence of other malignancies or severe concurrent infections.

### 2.10 Cell Culture and Cell Transfection

Human monocytes (THP‐1) cells (Cell Bank of the Chinese Academy of Sciences) were cultured in RPMI‐1640 + 10% FBS (Gibco; Thermo Fisher Scientific) and differentiated with 100 ng/mL phorbol‐12‐myristate‐13‐acetate (PMA, HY‐18739, MedChemExpress, USA) for 48 h. For TC2N overexpression, the full‐length open reading frame was cloned into pMSCV; lentivirus was packaged (Genomeditech, Shanghai, China), and cells were transfected using Lipofectamine 2000 (Invitrogen Preservation, Carlsbad, CA, USA), followed by Puromycin (Sigma) selection. The transfected THP‐1 macrophages were then treated with 50 μg/mL of ox‐LDL (Sigma) for 24 h.

### 2.11 Quantitative Real‐Time Polymerase Chain Reaction (RT‐qPCR) Analysis

Total RNA was extracted with TRIzol (Invitrogen, Carlsbad, CA), quantified by NanoDrop (A260/A280 1.8‐2.0; Thermo Fisher Scientific), and 1 μg was reverse‐transcribed with PrimeScript RT Master Mix (42°C 2 min, 37°C 15 min, 85°C 5 s; Takara). qPCR used SYBR Premix Ex Taq (Takara). Primer sequences are shown in Table S1. qPCR conditions: 95°C 10 min; 40 cycles of 94°C 2 min and 60°C 50 s; 60°C 1 min. Relative expression was determined by the 2^−ΔΔCt^ method with GAPDH normalization.

### 2.12 Western Blot (WB) Analysis

As previously reported (Ye et al. 2017), WB was performed. Cells were lysed in RIPA buffer (Sigma, MO, USA), and protein concentration was measured by BCA assay (Thermo Fisher Scientific). Equal amounts of protein were separated by SDS‐PAGE, transferred to polyvinylidene fluoride (PVDF) membranes, blocked with 5% non‐fat milk, and incubated overnight at 4°C with rabbit polyclonal TC2N antibody (1:500; Abcam). ACTIN was adopted as the loading control. After incubation with horseradish peroxidase (HRP)‐conjugated secondary antibody, signals were detected by enhanced chemiluminescence (ECL) chemiluminescence.

### 2.13 Enzyme‐Linked Immunosorbent Assay (ELISA)

The levels of tumor necrosis factor‐alpha (TNF‐α), interleukin‐1 beta (IL‐1β), and interleukin‐6 (IL‐6) were measured using ELISA kits (R&D, USA) according to the manufacturer's protocols. In brief, cell culture supernatants were added to pre‐coated wells, incubated with biotinylated detection antibody and HRP‐conjugated streptavidin, followed by tetramethylbenzidine (TMB) substrate development. Absorbance was read at 450 nm, and concentrations were determined from standard curves.

### 2.14 Oil Red O Staining

After phosphate buffered saline (PBS) washing and 10% formalin fixation, THP‐1 macrophages were pretreated with 60% isopropyl alcohol and stained using 0.2% Oil Red O (Sigma‐Aldrich) dissolved in the same alcohol. Images were captured by light microscopy at 100× magnification (scale bar: 500 μm).

### 2.15 Statistical Analysis

Data is presented as mean ± SD. Comparisons between two groups were performed using unpaired Student's *t*‐test, and comparisons among three or more groups were performed using one‐way ANOVA with Tukey's post hoc test. All statistical analyses were conducted using SPSS 16.0. A *p* < 0.05 was considered statistically significant.

## 3 Result

### 3.1 Identification of DEGs

**FIGURE 1:**
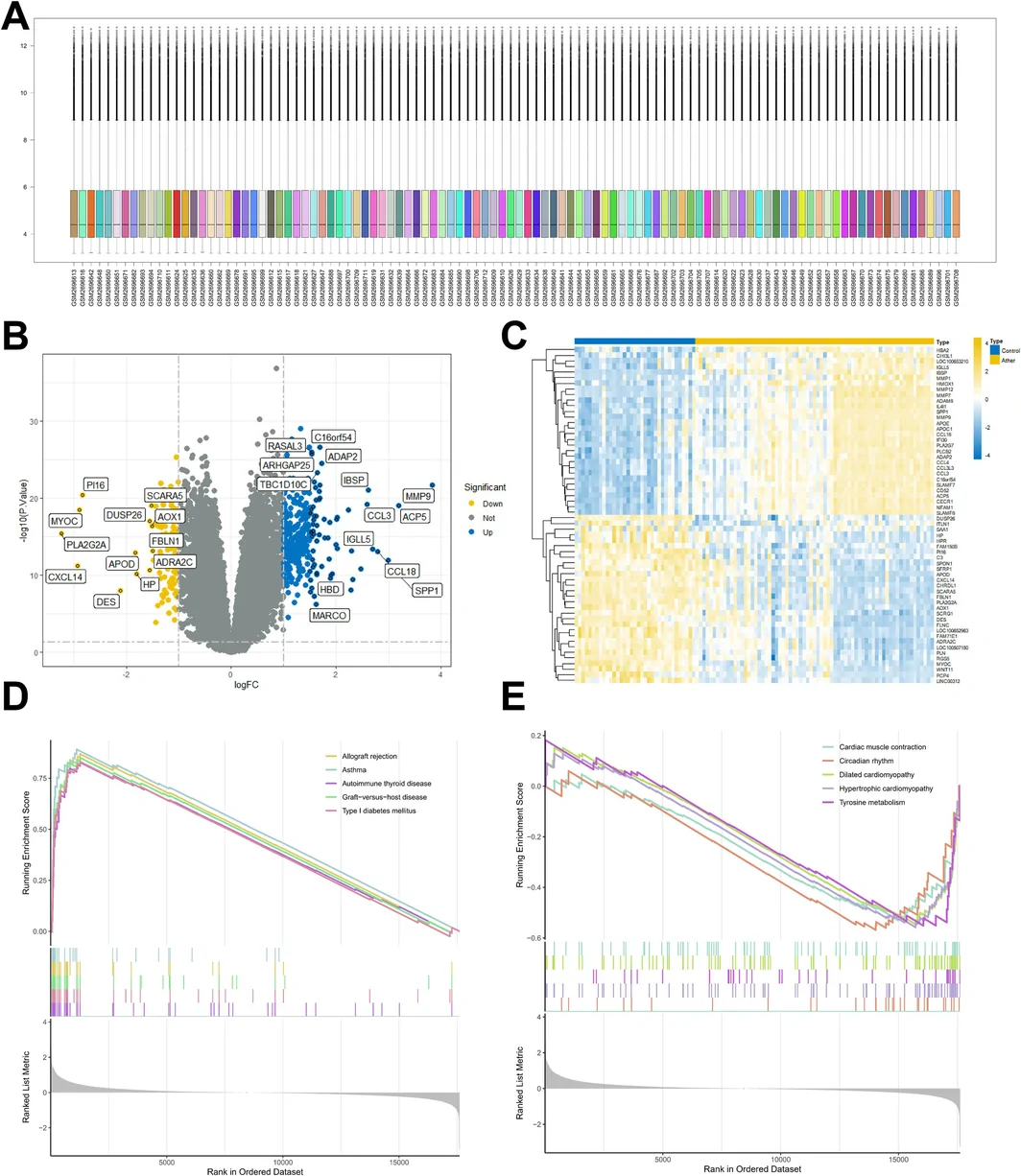
Screening of DEGs related to AS and analysis of GSEA pathway. (A) Normalization results of gene expression data after quality control. (B) The global expression difference distribution map of 418 DEGs in GSE100927 dataset. (C) The expression pattern heat map of the first 60 genes with the most significant differential expression between the two groups of samples. (D,E) The results of GSEA functional enrichment analysis of up‐regulated and down‐regulated genomes.

Following normalization (Figure 1A), the expression dataset was split into an AS patients' group and a control group for differential expression analysis through the limma package, and altogether 418 DEGs were found based on the pre—set criteria of |log_2_ fold change| ≥ 1 and an adjusted *p‐value* less than 0.05, including 295 genes which were highly activated and 123 genes which were highly inhibited (Figure 1B), and Figure 1C showed a heatmap demonstrating the expression patterns of the top 30 activated and top 30 inhibited DEGs among the samples, and then GSEA was carried out to clarify the functional enrichment situations of the activated and inhibited gene groups as shown in Figure 1D and Figure 1E correspondingly.

### 3.2 Construction of Co‐Expression Networks via WGCNA and Identification/Validation of the Hub Module (Blue Module)

**FIGURE 2:**
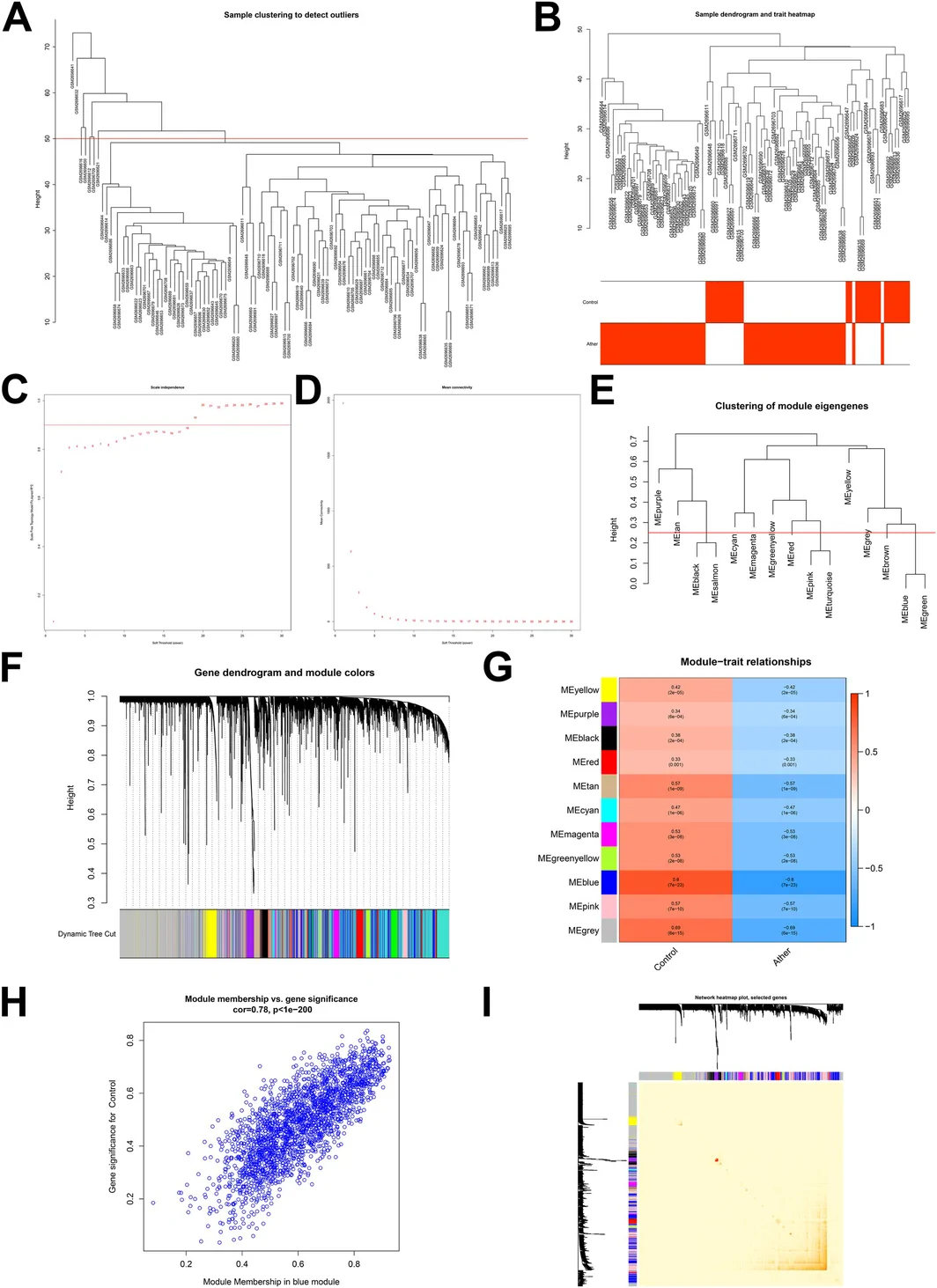
WGCNA‐based analysis and identification of key modules. (A) Sample clustering dendrogram: Removal of outlier samples, leaving 104 samples for analysis. (B) Sample dendrogram and trait heatmap showing AS and control group characteristics. (C) Assessment of soft‐thresholding power (*β*) based on scale‐free topology fit index *R* ^2^. (D) Relationship between soft‐thresholding power and mean connectivity (*β* = 18 selected). (E) Module merging via dynamic tree‐cutting (merge height = 0.25, minimum module size = 50 genes). (F) Clustering dendrogram of co‐expressed genes, identifying 11 distinct modules. (G) Module‐trait association analysis: Blue module showing the strongest correlation with AS. (H) Scatter plot of module membership (MM) versus gene significance (GS) in the blue module (*r* = 0.78, *p* < 1e‐200). (I) Heatmap of inter‐module connectivity: Low correlation between non‐adjacent modules, confirming module independence.

FPKM normalization was performed, and after sample quality filtering, 104 samples were retained (Figure 2A). WGCNA was then applied to construct a scale‐free co‐expression network, with a soft‐thresholding power of 18 selected to achieve a scale‐free topology fit (Figure 2C,D). This analysis identified 11 distinct co‐expression modules (Figure 2E,F). Module‐trait association analysis revealed that the blue module exhibited the strongest correlation with AS (Figure 2G), containing 2159 genes. Within this module, a high correlation between module membership and gene significance was observed (*r* = 0.78, *p* < 1e‐200, Figure 2H), confirming the strong relevance of this module to the AS trait (Figure 2I).

### 3.3 Enrichment and Differential Gene Screening of Aging‐Related Genes

**FIGURE 3:**
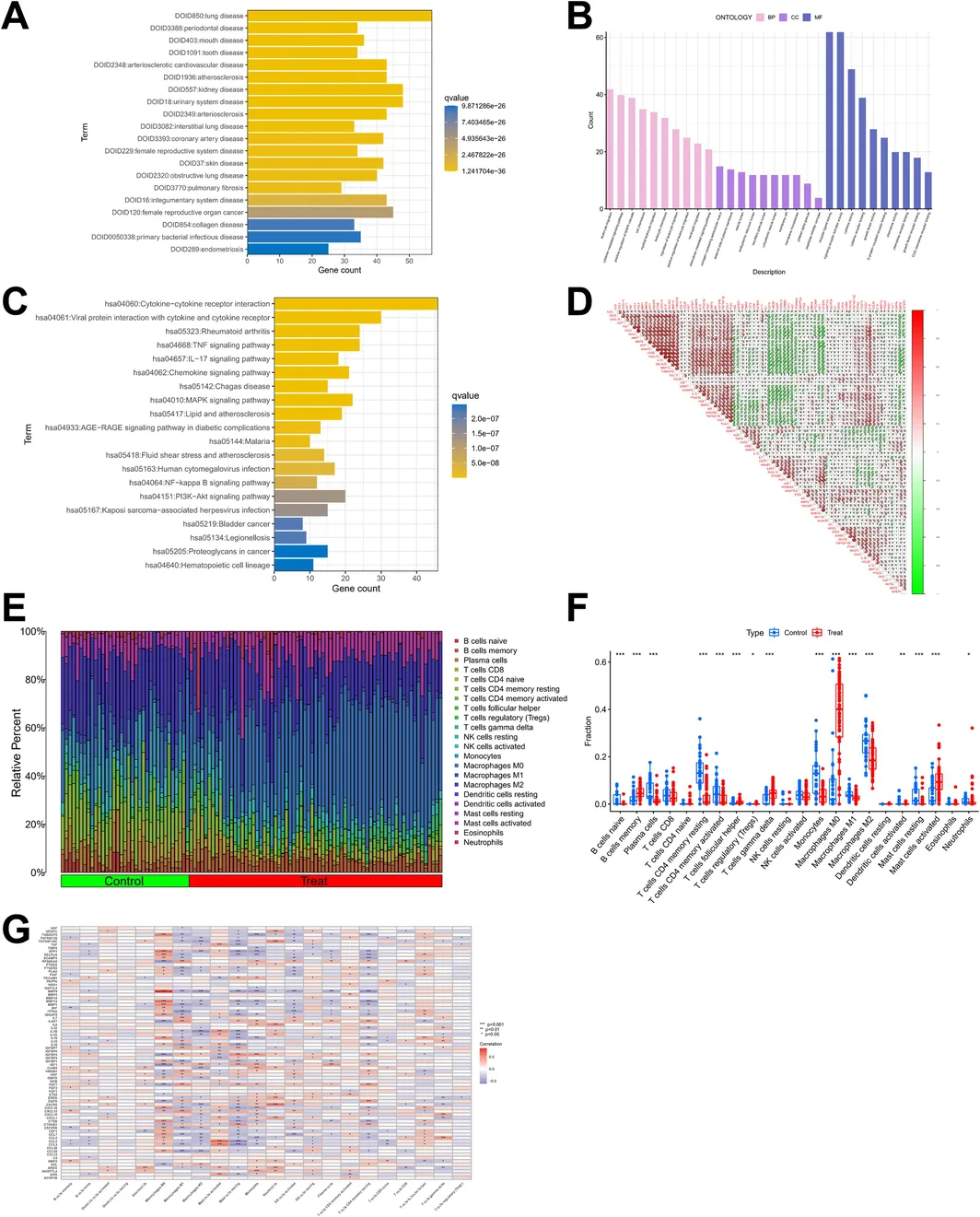
Multi‐level functional enrichment analysis, differential expression network, and immune infiltration correlation of aging‐related genes. (A) Disease Ontology (DO) enrichment analysis of aging‐related genes: Significant enrichment in atherosclerotic diseases. (B) GO Biological Process (BP) enrichment analysis of aging‐related genes. (C) KEGG pathway enrichment analysis of aging‐related genes. (D) Co‐expression correlation network of 76 aging‐related differentially expressed genes (DEGs). (E) A stacked histogram of 22 immune cells in the AS group and the control group. (F) Box plot of 22 immune cell infiltrations between AS and control groups. (G) Heat map of the correlation between 76 aging‐related DEGs and 22 immune cell infiltration. * represented the statistical *p*‐value (*p < 0.05; **p < 0.01; ***p < 0.001, ns, no significant).

The R software package was utilized for the enrichment and examination of 125 genes associated with aging and through GO enrichment analysis, it was found that these genes were considerably enriched in atherosclerotic diseases and immunomodulating pathways, especially those regulating leukocytes and neutrophils (Figure 3A,B). Kyoto Encyclopedia of Genes and Genomes (KEGG) analysis illustrated substantial connections with pathways linked to lipid metabolism and atherosclerosis, specifically Lipid and atherosclerosis and Fluid shear stress and atherosclerosis, as well as the aging‐associated AGE‐RAGE signaling pathway in diabetic complications, which directly links the accumulation of advanced glycation end‐products (AGEs) with age and AS (Figure 3C). Heatmap and box plot analyses showed that most of these genes were upregulated in the AS group compared with controls (Figure S1A,B). Differential expression analysis subsequently identified 76 aging‐related DEGs, and a co‐expression network among these DEGs was constructed (Figure 3D).

### 3.4 CIBERSORT‐Based Immune Cell Infiltration Analysis and Correlation Study Between Aging‐Related DEGs and Immune Cells

The enrichment study of DEGs and aging—associated genes within the GSE100927 dataset showed important immune—related processes so initially we measured the makeup of 22 types of immune cells across all samples through the CIBERSORT algorithm in R software and the outcomes were shown as stacked histograms (Figure 3E) and box plots (Figure 3F) with the stacked histograms illustrating the relative amounts of each immune cell type per sample (adding up to 1) and the box plots presenting the distribution of immune cell portions, the center line standing for the median and the box edges showing the interquartile range and when we analyzed these data we found that stimulated macrophages, gamma delta T cells, and mast cells were the main immune cell groups; after that we looked into the connections between 76 differently expressed aging—related genes and immune cell fractions by means of a heat map (Figure 3G) and it was worth noting that TUBGCP2, CXCL16, and MMP9 had positive relationships to M0 macrophages but negative ones to M1 and M2 macrophages which were especially meaningful considering the known function of macrophages as crucial immune mediators during the development of AS plaques (Lin et al. 2021) and the recent study indicating lower M2 macrophage levels in weak plaques (Zhang et al. 2024).

### 3.5 Aging Gene Signatures and Immune Microenvironment in AS Subtypes

**FIGURE 4:**
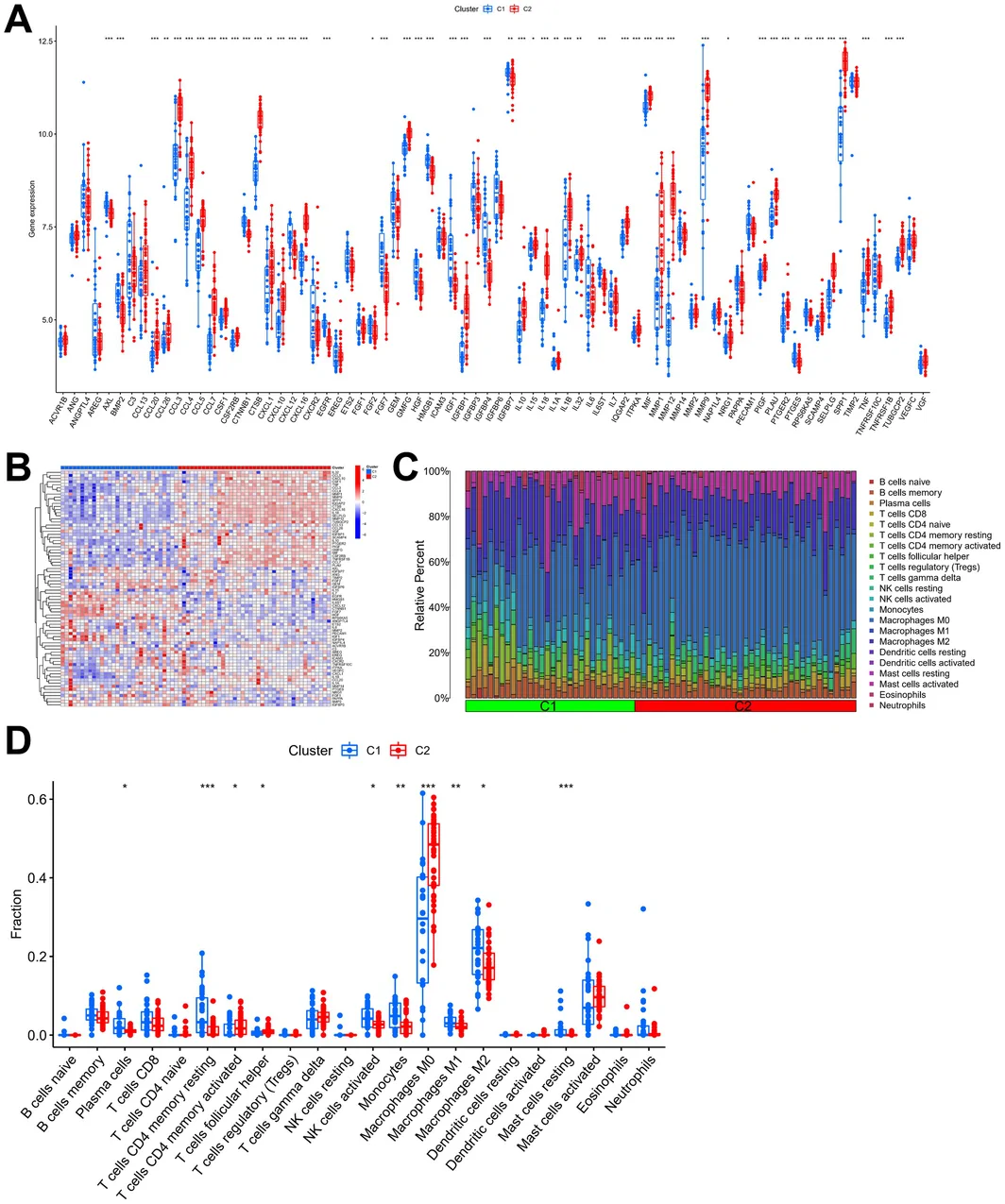
Expression characteristics of aging‐related DEGs and immune infiltration in AS subtypes. (A, B) The expression patterns of 76 aging‐related DEGs in C1 and C2 subtypes. (C) Stacked histogram of 22 immune cell compositions in C1 and C2 subtypes (based on CIBERSORT). (D) Box plot comparison of 22 immune cell infiltration levels between C1 and C2 subtypes. *p < 0.05, **p < 0.01, ***p < 0.001.

Consensus clustering of 69 AS samples identified two stable molecular subtypes (Subgroup I, *n* = 30; Subgroup II, *n* = 39), with high intra‐group homogeneity and inter‐group heterogeneity (Figure S2A–D). PCA further confirmed the clear transcriptional distinction between the two subtypes (Figure S2E). Consensus clustering identified two stable molecular subtypes (Subgroup I/C1, *n* = 30; Subgroup II/C2, *n* = 39), with significant differential expression of 76 aging‐related DEGs between the two subtypes (Figure 4A,B). Notably, IGF1, HMGB1, CXCL12, and AXL were positively associated with the C1 subtype. Immune infiltration analysis using CIBERSORT revealed that the C2 subtype was predominantly linked to immune‐related pathways, with chemokines (CCL3, CCL6, CCL13, CXCL10, CXCL16) and interleukins showing significant positive correlations, and macrophages exhibiting the strongest association across all AS subtypes (Figure 4C,D).

### 3.6 A 5‐Gene Nomogram Predictive Model for AS Based on Machine Learning

**FIGURE 5:**
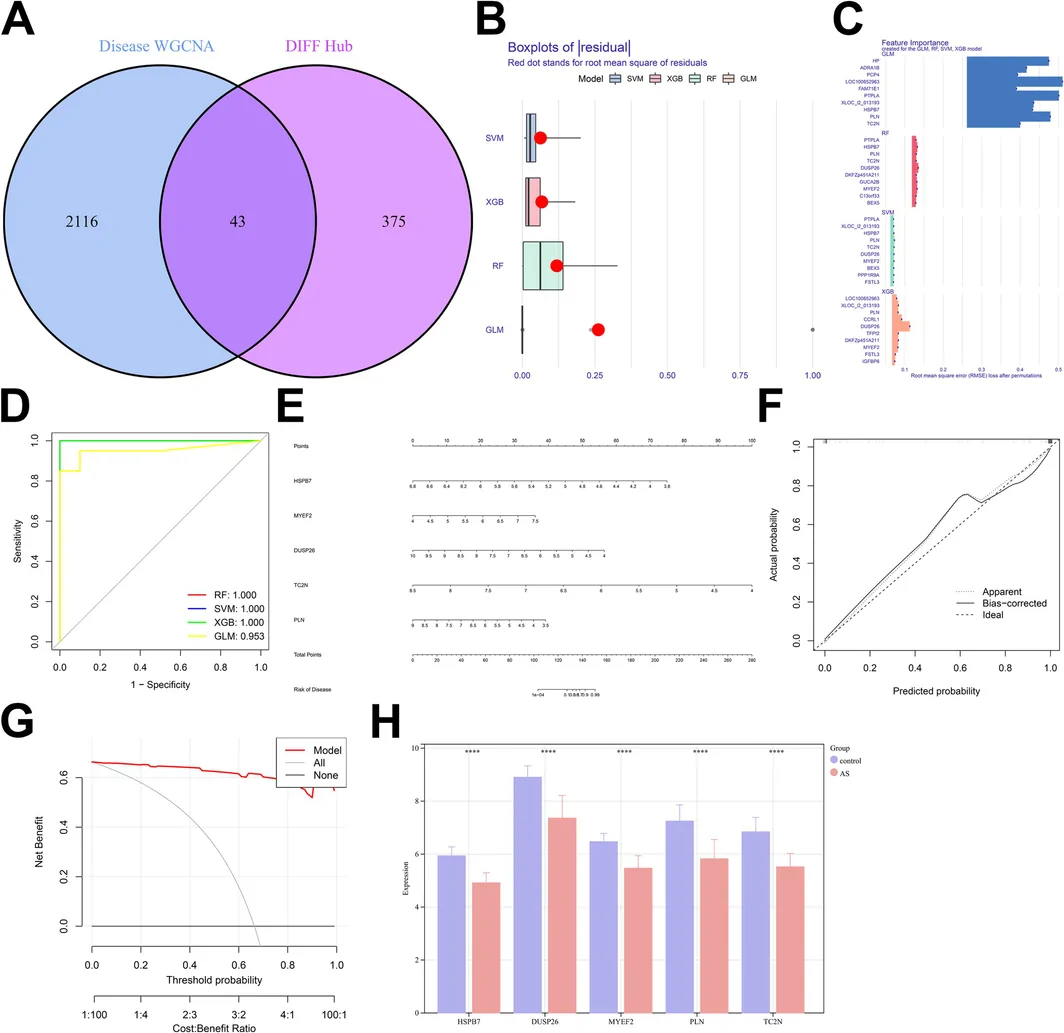
Screening of AS core genes, nomogram construction, and clinical utility evaluation based on multiple machine learning models. (A) Venn diagram showing 43 overlapping candidate hub genes identified by WGCNA and limma differential analysis. (B) Boxplot comparison of error rates among four machine learning models (SVM, RF, GLM, XGB). (C) Feature importance analysis of four models: XGB and SVM showing the highest contributions. (D) ROC curve comparison of four machine learning models (SVM, RF, GLM, XGB). (E) Nomogram prediction model based on five core genes (HSPB7, MYEF2, DUSP26, TC2N, PLN). (F) Calibration curve of the nomogram prediction model with bootstrap correction. (G) Decision curve analysis (DCA) of the nomogram model demonstrating good clinical utility. (H) Comparison of expression levels of five core genes in the GSE100927 dataset. ****p < 0.0001.

The intersection of key module genes determined via WGCNA and DEGs examined with limma resulted in 43 genes (Figure 5A). Four machine learning algorithms (SVM, RF, GLM, and XGB) were then applied for feature selection. Based on comprehensive performance evaluation (Figure 5B–D), SVM was identified as the optimal model, yielding five hub genes: TC2N, HSPB7, MYEF2, DUSP26, and PLN. A nomogram containing these five genes was developed (Figure 5E) and then evaluated with a calibration curve (Figure 5F). Finally, a Decision Curve Analysis (DCA) was carried out (Figure 5G) and it was shown that the model's red curves mostly occupied the upper area demonstrating strong clinical usefulness, Figure 5H presented a comparison of the expression levels of the five key genes throughout the cohort.

### 3.7 Single‐Cell RNA Sequencing Data Processing and Cell Type Identification

After standard quality control and PCA‐based dimension reduction (Figure S3). Unsupervised clustering at a resolution of 0.2 identified 14 distinct clusters (Figure S4A–C), and the top five marker genes for each cluster were determined (Figure S4D–E). Using the SingleR algorithm, the 14 clusters were annotated as chondrocytes, endothelial cells, macrophages, monocytes, B cells, smooth muscle cells, NK cells, and T cells (Figure S5A). The cellular composition varied markedly between AC and PA groups, with T cells and chondrocytes being the most abundant populations in both groups (Figure S5B). Trajectory analysis revealed a differentiation hierarchy among chondrocytes, with multiple distinct differentiation states identified (Figure S5C,D). Notably, chondrocyte differentiation appeared to be accelerated in the AC group compared with the control group (Figure S5E,F).

### 3.8 Verification of Hub Gene Single Cell Data

**FIGURE 6:**
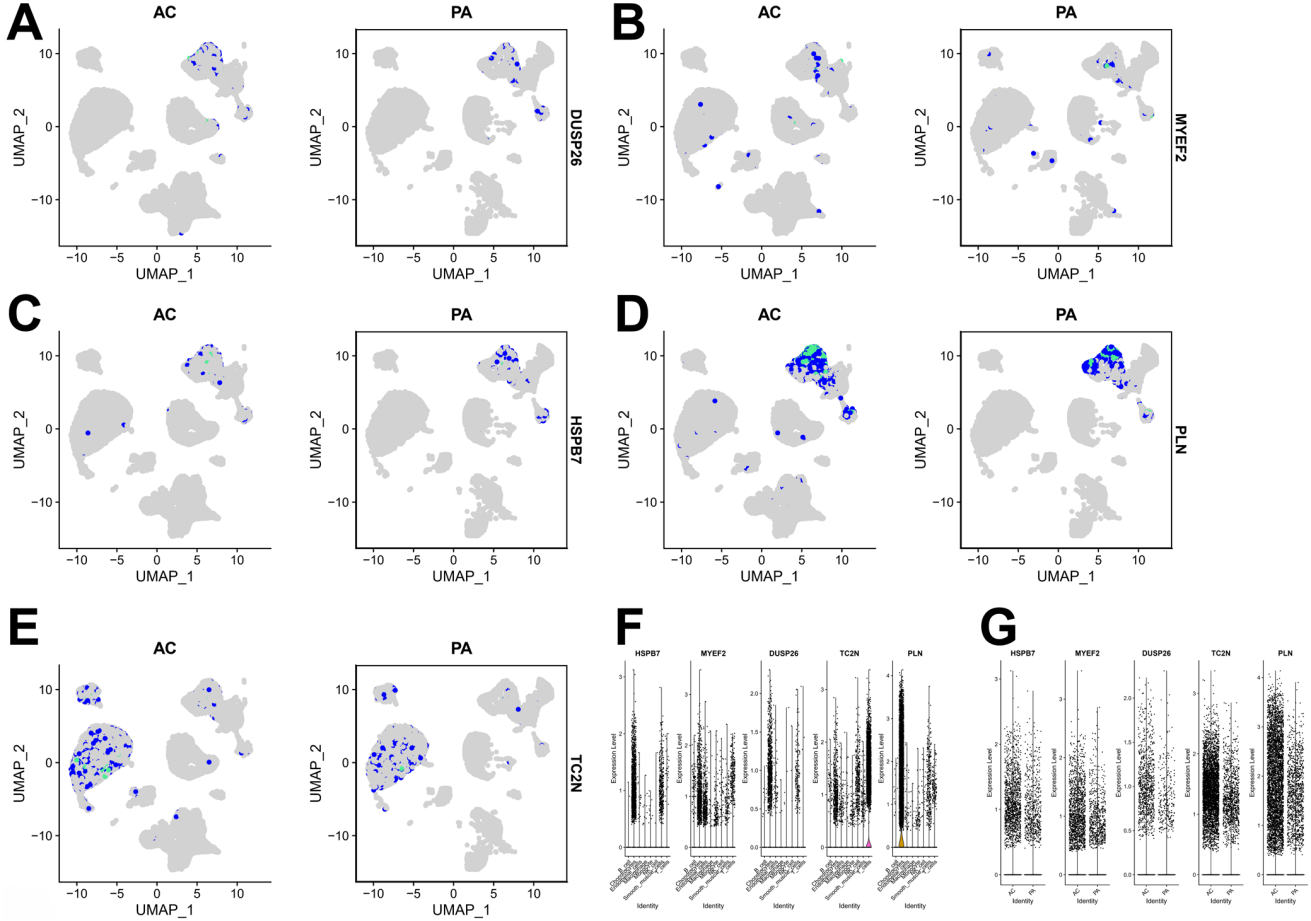
Validation of expression patterns and pseudotime expression trajectories of five core genes in single‐cell data. (A–E) UMAP plot of HSPB7, MYEF2, DUSP26, TC2N, and PLN expression distribution in AC and PA groups. (F) Expression density distribution of five core genes across different cell types, with highest expression in chondrocytes. (G) Comparison of five core gene expressions between AC and PA groups.

After finishing the single—cell data processing, the expression patterns of five crucial genes, namely HSPB7, MYEF2, DUSP26, TC2N, and PLN, were made visible by utilizing the Featureplot function to produce UMAP plots for both the AC and PA groups (Figure 6A–E), and it was worth noting that HSPB7, MYEF2, and PLN showed high expression in chondrocytes and smooth muscle cells but were not present in T cells as AS progressed. While the TC2N gene was mainly located in T cells and killer cells, there was a substantial decrease in its expression in T cells within atherosclerotic samples, and DUSP26 expression was mostly restricted to chondrocytes and endothelial cells, and it decreased significantly in endothelial cells from atherosclerotic samples. And Figure 6F presented the cell‐type‐specific expression density throughout the entire sample which verified the high expression of these central genes in chondrocytes, and Figure 6G further indicated that these genes were downregulated in the PA group when compared to the AC group which was in line with previous analytical results, and lastly, the expression changes of the five key genes across seven pseudo—temporal stages were examined (Figure S6).

### 3.9 TC2N and PLN Are Significantly Reduced in Atherosclerotic Plaque Tissue

**FIGURE 7:**
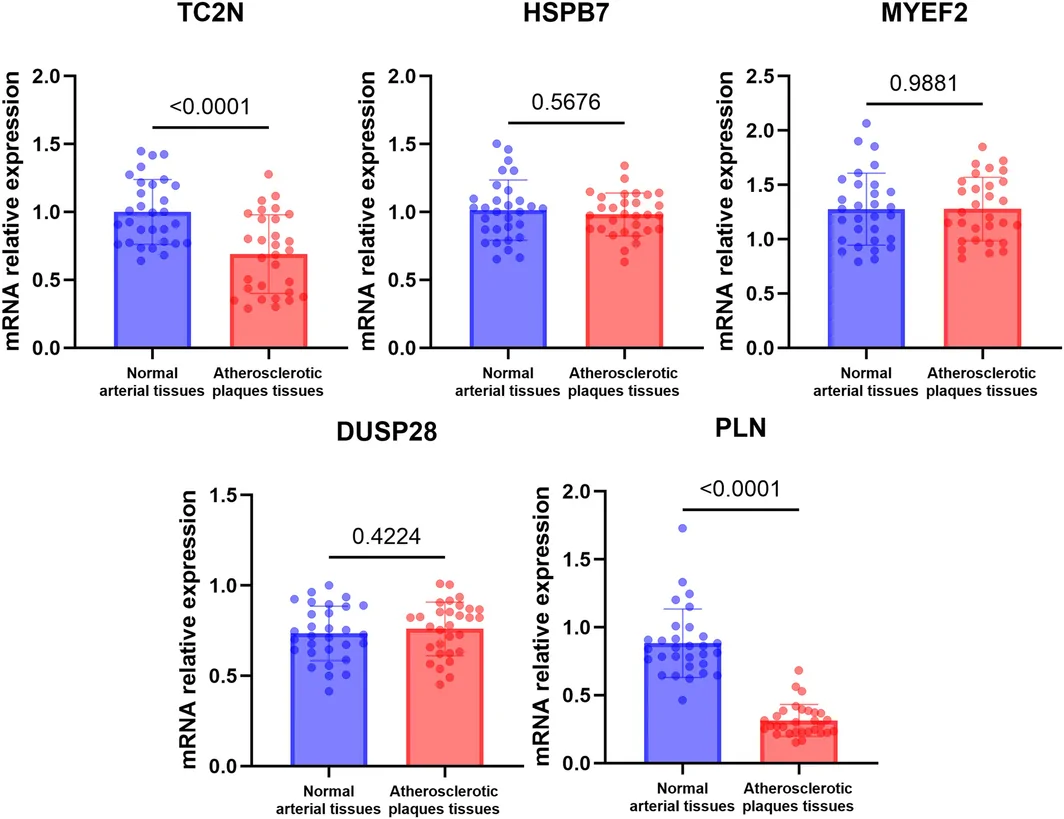
qPCR of TC2N, HSPB7, MYEF2, DUSP28, PLN in atherosclerotic plaques and normal arterial tissues of patients.

The expression levels of five crucial genes (HSPB7, MYEF2, DUSP26, TC2N, and PLN) in atherosclerotic plaques and adjacent normal arterial tissues were verified through experiments by means of qPCR and are shown in Figure 7. Compared with normal artery tissue, TC2N and PLN expression in atherosclerotic plaque tissue was significantly reduced.

### 3.10 TC2N Overexpression Inhibited Ox‐LDL‐Induced THP‐1 Macrophage Inflammatory Response and Foaming

**FIGURE 8:**
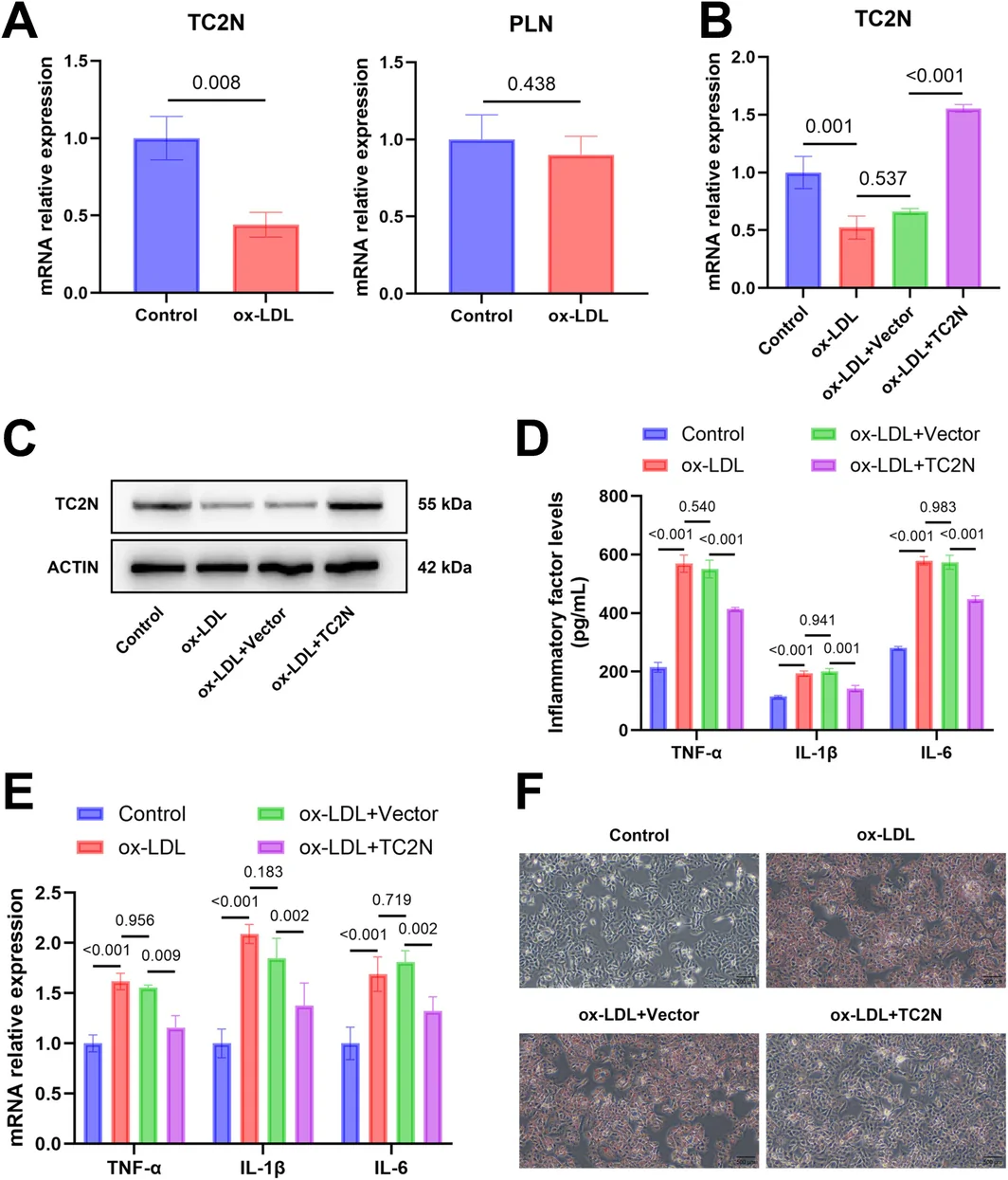
TC2N Overexpression Inhibited Ox‐LDL‐Induced THP‐1 Macrophage Inflammatory Response and Foaming. (A) Relative mRNA expression of TC2N and PLN in THP‐1 cells treated with ox‐LDL. (B, C) ox‐LDL induced THP‐1 macrophages with TC2N or vector stable transfection were identified by qRT‐PCR and WB. (D, E) Levels of inflammatory factors (TNF‐α, IL‐1β, and IL‐6) were detected via ELISA and qRT‐PCR. (F) ox‐LDL induced THP‐1 macrophages with TC2N or vector stable transfection were stained with Oil Red O (× 100, 500 μm).

Ox‐LDL (50 μg/mL) significantly reduced TC2N mRNA levels in THP‐1 macrophages (Figure 8A). TC2N‐overexpressing stable cells were established via lentiviral transduction (Figure 8B,C). TC2N overexpression suppressed ox‐LDL‐induced production of pro‐inflammatory cytokines (TNF‐α, IL‐1β, and IL‐6) (Figure 8D,E) and also reduced intracellular lipid accumulation, as shown by Oil Red O staining (Figure 8F). Thus, TC2N alleviates both the inflammatory response and foaming in ox‐LDL‐treated THP‐1 macrophages.

## 4 Discussion

Cardiovascular diseases (CVDs) pose a substantial public health burden in China, surpassing those of cancers and other major causes of death (Wang et al. 2023); atherosclerosis, the main underlying pathology of CVDs, the immune system is critically involved in starting and developing AS lesions (He et al. 2024); in the initial phase of AS, low‐density lipoprotein (LDL) gets stuck in the intima and changes through enzymatic and oxidative procedures to become danger‐associated molecular patterns (DAMPs), this alteration makes LDL immunogenic thus activating vascular endothelial cells and attracting different immune cells to the vascular wall, macrophages have various functions here from engulfing foreign substances to releasing immune active molecules and their characteristics and capabilities are influenced by environmental factors (Ijaz et al. 2025); cellular senescence has turned out to be a vital factor in age‐related diseases as senescent cells pile up in areas of tissue injury such as atherosclerotic plaques especially at arterial branch points and accordingly the targeted removal of senescent cells has been shown to reduce atherosclerotic plaque formation and ease cardiac aging (Lipscomb et al. 2025).

Recent research has emphasized the importance of age‐related genes in the development of diverse age‐linked diseases. For example, Hu et al. created a risk classification and predictive model for lung squamous cell carcinoma utilizing age‐related gene signatures (Hu et al. 2022). The growing accessibility of public genomic datasets has enabled extensive research on the connections between RNA‐seq data and clinical results; nevertheless, the function of age‐related genes in AS still hasn't been thoroughly examined. Aging is a natural and unavoidable process marked by functional deterioration and, from a pathophysiological point of view, metabolic disruption and weakened immune reactions are known risk factors for many chronic diseases as people grow older. In this study, we found 43 DEGs related to AS by overlapping the DEGs obtained from limma analysis and those selected through weighted gene WGCNA, then five key genes were identified using four different algorithms and the AS samples were divided according to age‐related gene expression profiles, differences in immune invasion among these subtypes were further studied and ultimately the expression patterns of the core genes were verified using single‐cell RNA sequencing analysis.

Functional enrichment analysis showed that the genes with differential expression were highly enriched in the mitogen‐activated protein kinase (MAPK) signaling pathway and human T‐cell leukemia virus type 1 (HTLV‐1) infection, osteoclast differentiation, and cytokine‐cytokine receptor interaction pathways, specifically, the upsurge in long non‐coding RNAs (lncRNAs) could regulate ERK phosphorylation, thus triggering inflammatory reactions via the MAPK pathway in atherosclerosis which eventually led to inflammation and collagen breakdown in AS plaques (Zhang et al. 2018), once chemokines and their receptors are imbalanced, they will promote the development of various diseases such as cancer, autoimmune diseases and chronic inflammation. Atherosclerosis is a chronic inflammation caused by the accumulation of lipids in the arterial wall, and chemokines and their receptors play an important role in the whole process of this disease (Srikanth et al. 2026; Zangas and Georgakis 2026), Picos et al. found that inflammation and oxidative stress were main instigators of atherosclerosis in the aging context (Picos et al. 2025), all these findings emphasized the crucial parts played by aging and immune malfunction in the origin and progress of atherosclerotic diseases, however, the immune—related functions of the five key genes and their connection with aging—related immune types needed further exploration.

Among the five central genes identified in this study, HSPB7 and PLN have been identified as cardiovascular related factors, with HSPB7 acting as a cardioprotective chaperone and PLN as a key regulator of cardiac SR calcium homeostasis (Wang et al. 2025; MacLennan and Kranias 2003). DUSP28 and MYEF2 are associated with a variety of pathological processes, including diabetes nephropathy, tumorigenesis and hepatocellular carcinoma, although their role in atherosclerosis has not been explored to a large extent (Lichtinger et al. 2012). However, the down‐regulation of these genes in atherosclerotic tissues observed in our bioinformatics and single cell analysis indicates that they may participate in the pathogenesis of AS, but their specific mechanism of action on the disease process deserves further study.

The Tac2–N (TC2N) is involved in human immune functioning (Hao et al. 2019); Hao et al.'s research showed that TC2N curbed the PI3K–AKT signal transduction by repressing P55γ and AKT phosphorylation, but although being related to immune regulation, its potential as a clinical immunotherapeutic target needed further verification (Hao et al. 2024). In line with these results, accumulating evidence has positioned TC2N as a potential diagnostic biomarker for AS. Zhang et al. identified TC2N as one of five hub genes associated with mitophagy and immune infiltration in AS, demonstrating strong diagnostic performance (AUC > 0.85) and confirming its differential expression in ox‐LDL‐treated macrophages (Zhang, Wang, et al. 2025). Our single–cell analysis found high TC2N expression in T cells in both AS and adjacent normal tissues, and we further observed that TC2N expression was significantly reduced in atherosclerotic plaque tissues from AS patients, as well as in ox‐LDL‐treated THP‐1 macrophages. Moreover, TC2N overexpression alleviated ox‐LDL‐induced inflammatory responses and cell foaming in THP‐1 macrophages, and these findings indicated that TC2N might be of diagnostic and therapeutic significance for diverse diseases, particularly in the context of chronic inflammatory conditions such as atherosclerosis. Collectively, these results suggest that TC2N may serve as a link between immune regulation and inflammatory vascular pathology.

Our research has several limitations. The expression of these crucial genes in the blood and tissues of AS patients was not experimentally verified. Although our screening outcomes were verified by means of atherosclerotic single—cell datasets, the results had to be interpreted carefully because of possible limitations related to the relatively small sample size (*n* = 30) for expression validation, which may reduce the statistical power for efficacy assessment, and gene expression instability and future research would center on carrying out ELISA experiments to validate the clinical utility of these five central genes within a bigger group. Finally, the working principle of these crucial genes in AS hasn't been verified via laboratory experiments and animal studies. We also note that the functional validation of TC2N was performed exclusively in the THP‐1 macrophage cell line; further studies using primary human macrophages or other relevant cell lines are warranted to confirm these findings. Further validation of its function is required in the in vivo model.

## 5 Conclusion

This study identified two aging‐related molecular subtypes with distinct immune landscapes and five hub genes associated with atherosclerosis progression. TC2N was further validated as a potential protective regulator of macrophage inflammation and foam cell formation. These findings provide an aging‐based molecular stratification framework and identify potential biomarkers and therapeutic targets for precision diagnosis and individualized treatment of atherosclerosis.

## Author Contributions

**Kang Chen:** conceptualization, data curation, investigation, formal analysis, writing – original draft. **Jibin Liu:** formal analysis, supervision, validation, visualization, writing – original draft. **Haixia Zhu:** conceptualization, visualization, supervision, writing – review and editing. **Qiyu Fan:** conceptualization, data curation, formal analysis, investigation, funding acquisition, writing – original draft. **Xun Diao:** data curation, investigation, project administration, software, writing – original draft. **Zhuopeng Xia:** conceptualization, formal analysis, validation, writing – review and editing. **Haizhong Yu:** methodology, project administration, resources, software, writing – review and editing.

## Funding

This work was supported by theNatural Science Foundation of Nanjing University of Chinese Medicine (XZR2024239) and Nantong University Clinical Medicine Special Program (226).

## Ethics Statement

Ethical approval for this study was granted by the Clinical Ethics Review Committee of Nantong Traditional Chinese Medicine Hospital (Approval No. 2025‐[004–3]), in accordance with the Declaration of Helsinki.

## Consent

Written informed consent was obtained from each participating patient.

## Conflicts of Interest

The authors declare no conflicts of interest.

## Supporting information


**Table S1:** PCR primer sequences.
**Figure S1:** The expression of senescence related genes in atherosclerosis group and control group. (A) Box plots display the expression of senescence related genes in two groups. (B) Senescence‐associated DEGs expression.
**Figure S2:** Consensus clustering analysis of gene expression profiles for AS samples. (A) Consensus score for different numbers of clusters for patients with atherosclerosis. (B) CDF plot displaying consensus distributions for each cluster (k) for patients with atherosclerosis. (C) Delta area plot reflecting the relative changes in the area under the CDF curve for atherosclerosis. (D) The heat map represents the consensus matrix with a cluster count of 2. (E) Visualizing the expression distribution of clusters by principal component analysis.
**Figure S3:** Single‐cell quality control and dimension reduction clustering. (A) Single‐cell quality control. (B) The proportion of mitochondrial and rRNA is adjusted to ensure the quality of cell samples. (C) Principle component analysis.
**Figure S4:** Single‐Cell Quality Control and Dimension Reduction Clustering (A–C) The AS dataset cells may be classified into 14 clusters. (D) The expression of the top five marker genes with the most prominence in each subgroup. (E) Heatmap showing the top DEGs (Wilcoxon test) in each cluster.
**Figure S5:** Single‐Cell Quality Control and Dimension Reduction Clustering. (A) Identify the cell type. (B) Histogram indicating the fraction of cell types in each sample. (C) Timing differences in cell differentiation. Darker blue represents an earlier stage of differentiation, while a lighter blue indicates a later stage of differentiation. (D) Seven stages of Chondrocytes differentiation. State 1 is the earliest stage of differentiation. (E) Differentiation of AS chondrocytes from normal chondrocytes. (F) All cells analyzed were chondrocytes.
**Figure S6:** Expression trajectories of five core genes across seven pseudotime stages.

## Data Availability

The data that support the findings of this study are available on request from the corresponding author. The data are not publicly available due to privacy or ethical restrictions.
