# Supplementary material for: Identification of Hub Gene Characteristics and Immune Landscapes Across Aging Subtypes in Atherosclerosis: A Machine Learning‐Based Multi‐Omics Study With Experimental Verification
Source: Chem Biol Drug Des. 2026 Aug 27;108(2):e70382. doi: 10.1111/cbdd.70382 (PMC13519764; doi:10.1111/cbdd.70382)
Supplement: Supplementary file 1 [file CBDD-108-e70382-s001.docx]

**Supplement Material**

**Table S1 PCR primer sequences**

| Gene | Forward | Reverse | Source |
| --- | --- | --- | --- |
| TC2N | AATGGCTCAGGATGCTCTGG | TCTGGCCTTTGCTTTTCGGA | human-derived |
| HSPB7 | GCAGGCAACATCAAGACCCT | CGCACCTCGATGTGGTTGTT |  |
| MYEF2 | GCGCTGGAGAAAAGAAGGGT | CCGCATCCTTAAAGAGCTCCA |  |
| DUSP28 | CCTTCCAGATGGTGAAGAGC | GGTGAATGTGGGTGACACTG |  |
| PLN | CCCCAGCTAAACACCCGTAA | AGCTGGCAGCCAAATATGAGA |  |
| TNF-α | AGAAAGCATGATCCGCGACG | TTTGCTACGACGTGGGCTAC |  |
| IL-1β | CCTGTGTGATGAAAGACGGC | TATGTCCCGACCATTGCTGT |  |
| IL-6 | GACAAAGCCAGAGTCCTTCAGAG | GGTCTTGGTCCTTAGCCACTC |  |
| GAPDH | CCATCTTCCAGGAGCGAGAT | TGCTGATGATCTTGAGGCTG |  |

**Fig. S1** The expression of senescence related genes in atherosclerosis group and control group. (A) Box plots display the expression of senescence related genes in two groups. (B) Senescence-associated DEGs expression.

**Fig. S2.** Consensus clustering analysis of gene expression profiles for AS samples. (A) Consensus score for different numbers of clusters for patients with atherosclerosis. (B) CDF plot displaying consensus distributions for each cluster (k) for patients with atherosclerosis. (C) Delta area plot reflecting the relative changes in the area under the CDF curve for atherosclerosis. (D) The heat map represents the consensus matrix with a cluster count of 2. (E) Visualizing the expression distribution of clusters by principal component analysis.

**Fig. S3.** Single-cell quality control and dimension reduction clustering. (A) Single-cell quality control. (B) The proportion of mitochondrial and rRNA is adjusted to ensure the quality of cell samples. (C) Principle component analysis.

**Fig. S4.** Single-Cell Quality Control and Dimension Reduction Clustering (A-C) The AS dataset cells may be classified into 14 clusters. (D) The expression of the top five marker genes with the most prominence in each subgroup. (E) Heatmap showing the top DEGs (Wilcoxon test) in each cluster.

**Fig. S5.** Single-Cell Quality Control and Dimension Reduction Clustering. (A) Identify the cell type. (B) Histogram indicating the fraction of cell types in each sample. (C) Timing differences in cell differentiation. Darker blue represents an earlier stage of differentiation, while a lighter blue indicates a later stage of differentiation. (D) Seven stages of Chondrocytes differentiation. State 1 is the earliest stage of differentiation. (E) Differentiation of AS chondrocytes from normal chondrocytes. (F) All cells analyzed were chondrocytes.

**Fig. S6.** Expression trajectories of five core genes across seven pseudotime stages.
